# Supplementary material for: Seed Storage Physiology of Lophomyrtus and Neomyrtus, Two Threatened Myrtaceae Genera Endemic to New Zealand
Source: Plants (Basel). 2023 Feb 27;12(5):1067. doi: 10.3390/plants12051067 (PMC10005796; doi:10.3390/plants12051067)
Supplement: Supplementary file 1 [file plants-12-01067-s001.zip › Supp_Data Table S1.pdf]

Supplementary Data Table S1: Cooling and warming thermodynamic properties of *Lophomyrtus bullata, L. obcordata* and *Neomyrtus pedunculata* seed following desiccation to various moisture contents. Different superscript letters within a column indicates significant difference at P < 0.05 based on a one-way ANOVA analysis (Tukey HSD test). Data are presented as mean ± SD (P < 0.05) for three different treatments.

Species	Moisture Content (g/g)	Sample Weight (mg)	Cooling				Warming			
			*Onset Temperature of Crystallization (°C)	**End Temperature of Crystallization (°C)	***Area of crystallization (mJ)	***Enthalpy of Melt (J/g)	*Onset Temperature of Melt (°C)	**End Temperature of Melt (°C)	***Area of Melt (mJ)	***Enthalpy of Melt (J/g)
L. bullata	0.053 ± 0.001	17 ± 3.9	–18.4 ± 1.4^{a}	–49.5 ± 2.8^{a}	7.3 ± 1.8^{a}	0.4 ± 0.9^{a}	–76.5 ± 0.8^{ab}	5.7 ± 0.8^{a}	48.9 ± 14.5^{a}	2.9 ± 0.6^{a}
	0.067 ± 0.001	16.4 ± 5.5	–19.7 ± 0.7^{ab}	–49.3 ± 1.6^{a}	7.3 ± 3.8^{a}	0.4 ± 0.1^{a}	–77.6 ± 0.4^{d}	3.0 ± 1.6^{abc}	68.7 ± 38.2^{a}	4.1 ± 2.1^{a}
	0.17 ±0.002	14.1 ± 3.3	–19.2 ± 2.0^{ab}	–47.0 ± 2.6^{a}	3.5 ± 1.2^{a}	0.3 ± 0.06^{a}	–76.4 ± 0.7^{ab}	4.8 ± 1.5^{ab}	37.2 ± 12.7^{a}	2.2 ± 0.5^{a}
L. obcordata	0.048 ± 0.001	13.1 ± 3.1	–23.2 ±3.6^{abc}	–50.8 ± 4.2^{b}	3.9 ± 2.8^{a}	0.3 ± 0.2^{a}	–77.1 ± 0.6^{abcd}	2.1 ± 2.5^{abc}	51.6 ± 8.8^{a}	4.2 ± 1.6^{a}
	0.06 ±0.001	19.3 ± 4.7	–23.7 ± 2.6^{abc}	–50.3 ± 0.4^{b}	6.3 ± 0.9^{a}	1.0 ± 0.9^{a}	–76.8 ± 0.4^{abc}	3.7 ± 0.4^{abc}	88.8 ± 48.5^{a}	4.6 ± 1.7^{a}
	0.12 ± 0.006	14.7 ± 2.6	–24.3 ±2.3^{bc}	–47.5 ± 5.9^{b}	4.8 ± 3.1^{a}	0.3 ± 0.1^{a}	–76.3 ± 0.6^{a}	2.2 ± 2.7^{abc}	32.3 ± 31.5^{a}	4.1 ± 2.0^{a}
N. pedunculata	0.069 ± 0.002	10.4 ± 0.6	–25.3 ± 0.5^{c}	–52.9 ± 1.0^{b}	7.2 ± 1.8^{a}	0.6 ± 0.1^{a}	–76.9 ± 0.7^{abcd}	0.15 ± 0.3^{c}	80.9 ± 49.7^{a}	7.6 ± 4.2^{a}
	0.084 ± 0.003	11.9 ± 0.4	–24.3 ± 0.6^{bc}	–51.3 ± 1.5^{b}	8.7 ± 2.1^{a}	0.7 ± 0.1^{a}	–77.6 ± 0.3^{cd}	1.2 ± 0.5^{bc}	72.1 ± 11.9^{a}	5.9 ± 0.9^{a}
	0.17 ± 0.004	10 ± 0.6	–25.4 ± 1.4^{c}	–52.6 ± 3.6^{b}	6.5 ± 1.9^{a}	0.6 ± 0.2^{a}	–77.3 ± 0.4^{bcd}	–0.3 ± 1.6^{c}	63.3 ± 11.9^{a}	6.2 ± 3.0^{a}
P value			*= 0.026*	*< 0.001*	*= 0.307*	*= 0.37*	*= 0.03*	*< 0.02*	*= 0.326*	*= 0.625*

* Onset temperature of the first peak that appeared during cooling or warming programmes
** End temperature of the last peak that appeared during cooling or warming programmes
*** Cumulative area / enthalpy of all the peaks that appeared during cooling or warming programmes
