# Supplementary material for: Seed Storage Physiology of Lophomyrtus and Neomyrtus, Two Threatened Myrtaceae Genera Endemic to New Zealand
Source: Plants (Basel). 2023 Feb 27;12(5):1067. doi: 10.3390/plants12051067 (PMC10005796; doi:10.3390/plants12051067)
Supplement: Supplementary file 1 [file plants-12-01067-s001.zip › Supp_Data Table S2.pdf]

Supplementary Data Table S2: Summary of the *Lophomyrtus bullata, L. obcordata, L. bullata x L. obcordata* and *Neomyrtus pedunculata* seed collected between April 2018 and June 2021

Species	Seed Lot	Region	Locality	GPS coordinates (DMS)	Elevation (m.a.s.l.)	Date Collected
L. bullata	Kap 19	Wellington	Maungakotukutuku valley	40°56'58.9"S 175°01'58.7"E	140	May 2019
L. bullata	Kap 21	Wellington	Maungakotukutuku valley	40°56'58.9"S 175°01'58.7"E	140	June 2021
L. bullata	Wrights	Wellington	Wright's Hill Reserve	41°17'34.7"S 174°44'02.3"E	301	June 2018
L. bullata	Butchers	Marlborough	Richmond Forest Park - Butchers Flat	41°17'47.65"S 173°20'29.54"E	210	April 2018
L. bullata x *obcordata*	Skyline 20	Wellington	Skyline Reserve	41°14'17.8"S 174°46'09.5"E	317	May 2020
L. obcordata	Ōtari 21	Wellington	Ōtari native botanic garden	41°16'04.7"S 174°45'23.7"E	101	May 2021
L. obcordata	Ōtari 19	Wellington	Ōtari native botanic garden	41°16'04.7"S 174°45'23.7"E	101	May 2019
L. obcordata	Old Mill Road	Marlborough	Nelson	41°23'22.4"S 173°35'54.63"E	87	April 2018
L. obcordata	Matai	Marlborough	Richmond Forest Park -	41°17'47.65"S 173°20'29.54"E	210	April 2018
N. pedunculata	Tar	Taranaki	Midhirst	39°17'43.7"S 174°11'11.3"E	502	March 2021
