# Supplementary material for: Seed Storage Physiology of Lophomyrtus and Neomyrtus, Two Threatened Myrtaceae Genera Endemic to New Zealand
Source: Plants (Basel). 2023 Feb 27;12(5):1067. doi: 10.3390/plants12051067 (PMC10005796; doi:10.3390/plants12051067)
Supplement: Supplementary file 1 [file plants-12-01067-s001.zip › Supp_Data Table S3.pdf]

Supplementary Data Table S3: Seed germination and dormancy breaking treatments for *Lophomyrtus bullata, Lophomyrtus obcordata, Lophomyrtus bullata x Lophomyrtus obcordata* and *Neomyrtus pedunculata*

**Species**	**Fresh**	**Dry stratification at 5°C for 4 weeks**	**Wet stratification at 5°C for 4 weeks**	**Wet stratification at 5°C for 20 weeks**
L. bullata	✔	✔	NT	NT
L. obcordata	✔	✔	NT	NT
L. bullata x L. obcordata	✔	✔	NT	NT
N. pedunculata	✔	✔	✔	✔

✔ indicates condition tested

NT indicates conditions not tested
