# Supplementary material for: Seed Storage Physiology of Lophomyrtus and Neomyrtus, Two Threatened Myrtaceae Genera Endemic to New Zealand
Source: Plants (Basel). 2023 Feb 27;12(5):1067. doi: 10.3390/plants12051067 (PMC10005796; doi:10.3390/plants12051067)
Supplement: Supplementary file 1 [file plants-12-01067-s001.zip › Supp_Data Table S4.pdf]

Supplementary Data Table S4: Seed storage trial for *Lophomyrtus bullata, L. obcordata* and L. bullata x obcordata seed desiccated to 15% equilibrated Relative Humidity (eRH) and stored at 5°C, –18°C and –196°C for 12 to 36 months. Non-desiccated *L. bullata* and *L. obcordata* seeds were also stored at 5°C for 24 months

Species	Identifier	Storage Temperature				Storage duration (months)
		Non-desiccated	Desiccated	Desiccated		
		5°C		–18°C	–196°C	
L. bullata	Kap 19	✔	✔	✔	✔	24
	Wrights	NT	NT	✔	NT	36
	Butchers	NT	NT	✔	NT	36
L. bullata x obcordata	Skyline 20	NT	NT	✔	✔	12
L. obcordata	Ōtari 19	✔	✔	✔	NT	24
	Old Mill Road	NT	NT	✔	NT	36
	Matai	NT	NT	✔	NT	36

✔ indicates condition tested

NT indicates conditions not tested
